# Association Analysis of Canonical Wnt Signalling Genes in Diabetic Nephropathy

**DOI:** 10.1371/journal.pone.0023904

**Published:** 2011-08-18

**Authors:** David H. Kavanagh, David A. Savage, Christopher C. Patterson, Amy Jayne McKnight, John K. Crean, Alexander P. Maxwell, Gareth J. McKay, the Warren 3/UK GoKinD Study Group

**Affiliations:** 1 Nephrology Research Group, Centre for Public Health, Queen's University Belfast, Belfast, United Kingdom; 2 Histocompatibility and Immunogenetics Laboratory, Blood Transfusion Service, Belfast City Hospital, Belfast, United Kingdom; 3 Epidemiology Research Group, Centre for Public Health, Queen's University Belfast, Belfast, United Kingdom; 4 Conway Institute, University College Dublin, Dublin, Ireland; ¶ Membership of The Warren 3/UK GoKinD Study Group is provided in the Acknowledgments.; University of Tor Vergata, Italy

## Abstract

**Aims/Hypothesis:**

Several studies have provided compelling evidence implicating the Wnt signalling pathway in the pathogenesis of diabetic nephropathy. Gene expression profiles associated with renal fibrosis have been attenuated through Wnt pathway modulation in model systems implicating Wnt pathway members as potential therapeutic targets for the treatment of diabetic nephropathy. We assessed tag and potentially functional single nucleotide polymorphisms (SNPs; n = 31) in four key Wnt pathway genes (*CTNNB1, AXIN2, LRP5* and *LRP6*) for association with diabetic nephropathy using a case-control design.

**Methods:**

SNPs were genotyped using Sequenom or Taqman technologies in 1351 individuals with type 1 diabetes (651 cases with nephropathy and 700 controls without nephropathy). Cases and controls were white and recruited from the UK and Ireland. Association analyses were performed using PLINK, to compare allele and haplotype frequencies in cases and controls. Adjustment for multiple testing was performed by permutation testing.

**Results:**

Following logistic regression analysis adjusted by collection centre, duration of T1D, and average HbA1c as covariates, a single SNP in *LRP6* (rs1337791) was significantly associated with DN (OR = 0.74; CI: 0.57–0.97; P = 0.028), although this was not maintained following correction for multiple testing. Three additional SNPs (rs2075241 in *LRP6*; rs3736228 and rs491347 both in *LRP5*) were marginally associated with diabetic nephropathy, but none of the associations were replicated in an independent dataset. Haplotype and subgroup analysis (according to duration of diabetes, and end-stage renal disease) also failed to reveal an association with diabetic nephropathy.

**Conclusions/Interpretation:**

Our results suggest that analysed common variants in *CTNNB1, AXIN2, LRP5* and *LRP6* are not strongly associated with diabetic nephropathy in type 1 diabetes among white individuals. Our findings, however, cannot entirely exclude these genes or other members of the Wnt pathway, from involvement in the pathogenesis of diabetic nephropathy as our study had limited power to detect variants with small effect size.

## Introduction

**Figure 1:**
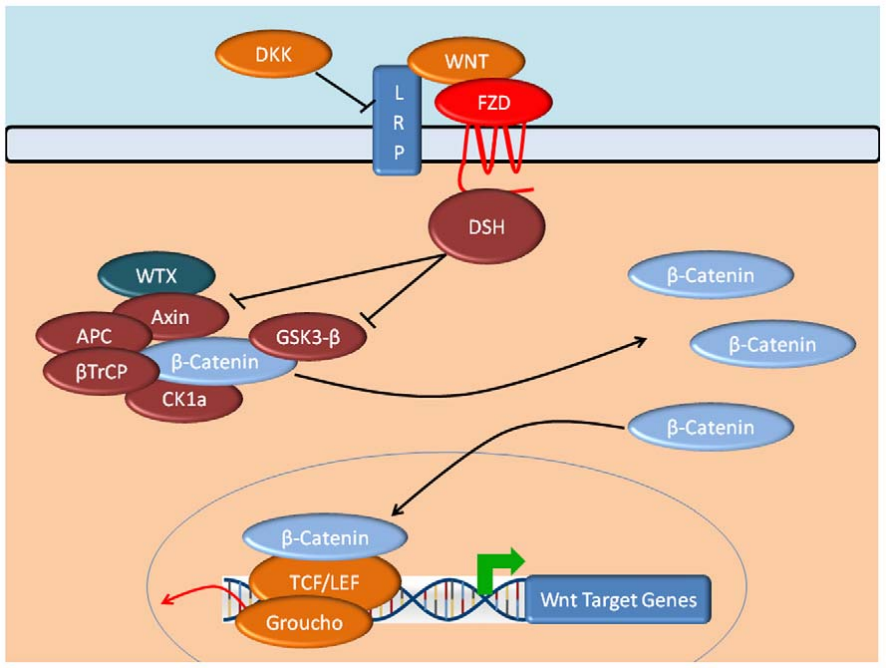
Schematic representation of the canonical Wnt signalling pathway. Gene names represented: Dishevelled (*DSH*), *adenomatous polyposis coli* (*APC*), low-density lipoprotein receptor related proteins (*LRP*), casein kinase 1 (*CK1a*), glycogen synthase kinase 3 (*GSK3- β*), β-transducin repeat-containing protein (*β-TrCP*), T-cell factor (*TCF*)/lymphoid enhancer factor (*LEF*), Wilms tumor-suppressor (*WTX*), Frizzled (*FZD*), dickkopf homolog 1.

Renal interstitial fibrosis and glomerular sclerosis are hallmarks of diabetic nephropathy (DN) and several studies have implicated the canonical Wnt pathway in this fibrotic process [1]–[2]. The Wnt/β-catenin signalling pathway modulates numerous developmental processes and mutations in Wnt/β-catenin pathway members have been implicated in multiple diseases [3]. In humans, 19 different specific Wnt ligands have been reported to induce Wnt/β-catenin signalling [3]. These ligands bind to transmembrane Frizzled (FZD) receptors and their co-receptors, low-density lipoprotein receptor related proteins 5 or 6 (LRP5/LRP6), forming a binding complex that recruits intracellular scaffolding protein Dishevelled (Figure 1). This leads to phosphorylation and sequestration of the Axin complex, which is composed of the proto-oncogene *adenomatous polyposis coli*, casein kinase 1 and glycogen synthase kinase 3, as well as the Axin protein itself. In the absence of Wnt, the Axin complex phosphorylates the cytoplasmic transcriptional coactivator β-catenin. This leads to subsequent recognition by β-transducin repeat-containing protein, which when ubiquitinated, promotes proteosomal degradation of β-catenin. When the Axin complex and Wnt ligands combine, β-catenin phosphorylation and ubiquitination ceases. The resultant stabilisation of intracellular β-catenin facilitates its translocation from the cytoplasm to the nucleus, where it interacts with transcription factors of the T-cell factor/lymphoid enhancer-binding factor family, initiating transcription of Wnt-responsive genes [3].

TGFβ1-induced epithelial-to-mesenchymal cell transition (EMT) promotes renal fibrosis [4], a characteristic pathological feature of DN. TGFβ1, integrin-linked kinase and Wnt pathways converge upon activation of β-catenin and EMT is initiated, implicating β-catenin as a master controller of multiple pathways. Furthermore, membrane bound E-cadherin/β-catenin complexes, which form the cell-to-cell junctions typical of an epithelial cell type, are lost during TGF-β1 and IGF-I induced EMT [5]. In addition, inhibition of GSK3β has been shown to prevent mesenchymal transition in human embryonic stem cells [6]. It is estimated that there are more than 1800 Wnt pathway gene targets [7] including transcription factor c-Myc, matrix metalloprotease MMP-7, endothelin-1, and fibronectin (a marker of fibrosis) [8]. Increased expression of Wnt-4 is associated with the deposition of fibronectin [9].

Variation in expression profiles of many Wnt ligands, FZD receptors and β-catenin have been reported in the unilateral ureteral obstruction (UUO) mouse model of renal injury and reduction in interstitial injury identified in response to dickkopf homolog 1 (DKK-1), a Wnt signalling antagonist [1]. Independently, DKK-1 was also shown to promote hyperglycaemia-induced mesangial matrix accumulation and renal dysfunction in rat mesangial cells [2]. Comparison of injury models is difficult; nonetheless existing evidence implicates Wnt signalling in the pathology of DN.

Our study was designed to assess association of common single nucleotide polymorphisms (SNPs) in four key genes of the canonical Wnt/β-catenin signalling pathway (*AXIN2, CTNNB1, LRP5* and *LRP6*) with DN using a case-control design involving 1467 individuals with type 1 diabetes. These genes were selected on the basis of their functional significance as key components of the Wnt signalling pathway and from differential expression profiles derived from human kidney biopsy material from DN cases compared to no nephropathy control samples. *CTNNB1* encodes the β-catenin protein, the major effector of the pathway responsible for transducing the Wnt activated signal from the cytoplasm to the nucleus and subsequent transcriptional activation of Wnt responsive genes. Both *LRP5* and *LRP6* are co-receptors specific to the canonical Wnt pathway enabling detection of the Wnt ligands. *AXIN2* is a key component of the destruction complex, which in the absence of a Wnt ligand, marks β-catenin for ubiquitin-dependent proteolytic degradation, thus preventing its translocation from the cytoplasm to the nucleus and subsequent transcriptional activation.

## Methods

### Participants

Ethics approval was obtained from the appropriate Research Ethics Committees, and written informed consent obtained prior to participation. All recruited individuals were white, had type 1 diabetes mellitus (T1D) diagnosed before 32 years of age and were born in the UK or Ireland. Patients (n = 718) and controls (n = 749) were from the Warren 3/UK Genetics of Kidneys in Diabetes (GoKinD) and all-Ireland collections [10]. The definition of DN in cases was based on development of persistent proteinuria (>0.5 g protein/24 h) at least 10 years after diagnosis of T1D, hypertension (blood pressure>135/85 mmHg or treatment with antihypertensive agents) and associated diabetic retinopathy. Controls were individuals with T1D for at least 15 years with normal urinary albumin excretion rates and no evidence of microalbuminuria on repeated testing. In addition, control subjects had not been prescribed antihypertensive drug treatment avoiding possible misclassification of diabetic individuals as ‘control phenotypes’ when the use of antihypertensive treatment may have reduced urinary albumin excretion into the normal range. Individuals with microalbuminuria were excluded from either case or control groups since it is not possible to confidently assign a case or control definition upon such individuals with microalbuminuria as their urinary albumin excretion may either regress or progress over time [11].

### SNP selection and genotyping

Tag SNPs for each gene investigated were selected from Phase III, release 2 HapMap (http://www.hapmap.org) CEPH data (Utah residents with ancestry in northern and western Europe; CEU) using Haploview (http://www.broadinstitute.org/haploview) to visualise linkage disequilibrium. Tag SNPs were selected using multimarker tagging where r2>0.8 (LOD threshold 3.0) for all downloaded SNPs with a minor allele frequency (MAF) ≥5%, genotype call rate ≥95%, and no deviation from Hardy–Weinberg equilibrium (HWE). Additional potentially functional SNPs were identified using the Ensembl genome browser (www.ensembl.org).

Genotyping was performed by MassARRAY iPLEX (Sequenom, San Diego, CA, USA) or Taqman 5′ nuclease (Applied Biosystems, Foster City, CA, USA) assays according to the manufacturers' instructions. Quality filters for exclusion of SNPs included call rates below 95% and deviation from HWE (P<0.001). DNA samples were excluded if missing genotypes exceeded 10%. Other quality control measures included parent/offspring trio samples, duplicates on plates, random sample allocation to plates, independent scoring of problematic genotypes by two individuals and re-sequencing of selected DNAs to validate genotypes.

### Statistical analysis

Clinical characteristics of cases and controls were compared using the z-test for large independent samples and the χ^2^ test. Association analyses were performed using PLINK (version 1.07; http://pngu.mgh.harvard.edu/~purcell/plink/). Initially a χ^2^ test for trend (1 *df*) was used with stratification by collection centre. Logistic regression analysis was performed on each SNP with terms for potential confounders (collection centre, sex, duration of T1D and HbA1c) included in the model. The level of statistical significance was set at 5% and adjustment for multiple testing performed by permutation test (n = 100,000). *In silico* replication of the most significant SNPs was sought through data extracted from the US Genetics of Kidneys in Diabetes (US GoKinD) study [12] available on dbGAP (http://www.ncbi.nlm.nih.gov/gap, dataset phs000018.v2) which was based on a stratified analysis of 935 DN cases and 944 no nephropathy controls. The US GoKinD genotyping was performed on the Affymetrix 5.0 SNP array (Affymetrix, Santa Clara, CA, USA). Although only one of the four most significant SNPs identified from this study was genotyped directly on this platform, surrogate markers in high LD based on 1000 Genomes pilot data (http://www.1000genomes.org/), were used as a proxies for the other three. Potential gene-gene interactions between the most significant SNPs were assessed using likelihood ratio χ^2^ tests in the logistic regression.

## Results

The clinical characteristics of the DN cases (n = 651) and diabetic controls (n = 700) genotyped in this study which met quality control filters are listed in Table 1. The average genotyping rate was 98.6%. There were more males, higher mean HbA1c and blood pressure values (despite the use of antihypertensive treatment) in the case group compared with the control group. All comparisons were significant at *P*<0.001 with the exception of age at diagnosis which did not differ significantly between groups. Approximately one quarter of cases (25.4%) had end-stage renal disease (ESRD).

**Table 1:** Clinical characteristics of diabetic nephropathy (DN) cases and diabetic controls.

Characteristic	DN cases(n = 651)	Controls(n = 700)
Male; n (%)	374 (57.5%)	299 (42.7%)
Age at diagnosis of T1D (yr)	14.7±7.6	15.4±7.9
Duration of T1D (yr)^a^	33.2±9.3	28.0±8.9
HbA1c (%)^b^	9.1±1.9	8.6±1.5
Systolic blood pressure (mmHg)^b^	145.0±21.0	124.9±14.4
Diastolic blood pressure (mmHg)^b^	81.7±11.5	75.4±7.8
Body mass index (kg/m^2^)	26.4±4.8	26.2±4.2
Serum cholesterol (mmol/L)	5.35±1.25	5.09±0.92
Serum creatinine (µmol/L);^c^ median (interquartile range)	130 (103–183)	91 (77–105)
Glomerular filtration rate (ml/min/1.73m^2^);^ c^median (interquartile range)	48 (34–66)	70 (60–87)
End-stage renal disease n (%)	165 (25.3%)	NA

Unless otherwise stated values are mean ± standard deviation.

a Calculated from the dates of diagnosis and recruitment.

b Average of the three most recent values prior to recruitment.

c Excludes subjects receiving renal replacement therapy (dialysis or transplant).

*P*<0.05 for age at diagnosis; *P*<0.001 for all other comparisons except body mass index.

We excluded 116 samples (67 patients and 49 controls) from the analysis with ≥10% missing genotypes. A total of 31 SNPs were genotyped, 28 using MassARRAY iPLEX technology, and 3 SNPs by Taqman 5′ nuclease assay in 651 cases and 700 controls (Table 2). The average call rate for all SNPs analysed was 98.65%. The genotype distribution for each SNP did not deviate significantly from HWE in either cases or controls. No duplicate or Mendelian inconsistencies were observed.

**Table 2:** Minor allele frequencies (MAF) and genotype counts in cases and controls.

			Case		Control			Confidence	
Gene	SNP	aAlleles	Counts	MAF	Counts	MAF	bOR	Interval	cP
*LRP6*	rs10466849	[T/C]	23/143/480	0.15	17/200/479	0.17	0.88	0.69–1.12	0.301
*LRP5*	rs11228202	[T/C]	15/146/489	0.14	9/159/531	0.13	1.10	0.84–1.44	0.486
*CTNNB1*	rs11564465	[T/C]	144/297/210	0.45	171/315/213	0.47	0.93	0.78–1.10	0.394
*LRP5*	rs11823032	[A/G]	53/276/288	0.31	72/297/316	0.32	0.91	0.74–1.10	0.324
*AXIN2*	rs11868547	[C/G]	140/336/175	0.47	158/331/209	0.46	1.00	0.84–1.19	0.981
*AXIN2*	rs12452196	[A/G]	12/160/479	0.14	15/165/519	0.14	1.11	0.86–1.43	0.425
*LRP6*	rs13377971	[A/G]	15/108/525	0.11	13/157/529	0.13	0.74	0.57–0.97	0.028
*LRP6*	rs2075241	[C/G]	30/192/429	0.19	17/173/510	0.15	1.24	0.98–1.56	0.075
*AXIN2*	rs2240308	[G/A]	167/304/178	0.49	161/349/187	0.48	1.06	0.89–1.26	0.53
*LRP5*	rs2242340	[T/C]	7/140/478	0.12	8/155/498	0.13	0.96	0.73–1.27	0.773
*LRP6*	rs2300230	[C/T]	4/78/564	0.07	5/85/609	0.07	1.20	0.84–1.71	0.318
*LRP6*	rs2302685	[C/T]	19/208/403	0.20	22/207/441	0.19	1.05	0.83–1.33	0.663
*LRP6*	rs2417085	[C/T]	153/282/166	0.49	142/327/201	0.46	1.03	0.86–1.24	0.742
*LRP5*	rs312014	[C/G]	79/312/260	0.36	92/289/318	0.34	1.10	0.91–1.32	0.335
*LRP5*	rs312016	[A/G]	49/273/329	0.28	59/246/394	0.26	1.11	0.91–1.35	0.297
*LRP5*	rs3736228	[T/C]	14/180/437	0.16	11/166/509	0.14	1.27	0.98–1.64	0.066
*LRP6*	rs3741792	[T/C]	4/72/573	0.06	4/82/612	0.06	1.22	0.85–1.76	0.28
*LRP5*	rs3781600	[C/G]	12/117/522	0.11	4/127/569	0.10	1.15	0.86–1.55	0.346
*AXIN2*	rs3923086	[T/G]	136/297/217	0.44	140/331/229	0.44	1.06	0.89–1.26	0.492
*AXIN2*	rs4074947	[T/C]	28/214/409	0.21	23/250/427	0.21	0.89	0.71–1.11	0.29
*AXIN2*	rs4128941	[A/G]	0/58/581	0.05	1/55/628	0.04	1.14	0.73–1.77	0.569
*AXIN2*	rs4541111	[T/G]	162/314/171	0.49	189/320/185	0.50	0.89	0.75–1.06	0.206
*AXIN2*	rs4791171	[A/G]	53/286/310	0.30	67/285/348	0.30	1.03	0.85–1.25	0.77
*LRP5*	rs491347	[C/T]	38/285/325	0.28	35/272/391	0.24	1.22	0.99–1.51	0.062
*LRP5*	rs4930573	[G/C]	35/238/378	0.24	34/249/417	0.23	1.05	0.85–1.30	0.669
*LRP5*	rs587397	[G/C]	2/109/540	0.09	12/106/582	0.09	0.96	0.71–1.31	0.804
*AXIN2*	rs7224837	[G/A]	6/126/491	0.11	12/145/515	0.13	1.01	0.76–1.34	0.96
*LRP6*	rs7305037	[C/T]	119/312/191	0.44	149/329/192	0.47	0.99	0.82–1.19	0.903
*AXIN2*	rs740026	[A/G]	128/307/212	0.44	122/356/215	0.43	1.00	0.83–1.20	0.996
*LRP5*	rs74744	[C/T]	110/313/228	0.41	125/349/226	0.43	0.90	0.75–1.08	0.258
*AXIN2*	rs757558	[T/G]	25/155/443	0.16	18/162/470	0.15	1.20	0.94–1.53	0.142

a Minor alleles are presented first followed by major allele.

b Odds ratios and 95% confidence intervals are calculated on a per allele basis for the first-mentioned allele assuming an additive model.

c P values were calculated as tests for trend (1 df) across genotypes and are adjusted by centre, gender, duration of disease and HbA1c level. Associations were no longer significant after adjustment for multiple testing performed by permutation test (n = 100,000).

Single marker testing stratified by collection centre identified one SNP (rs1337791 in *LRP6*) significantly associated and three SNPs (rs2075241 in *LRP6*; rs3736228 and rs491347 both in *LRP5*) marginally associated with DN. Following logistic regression analysis adjusted by collection centre, duration of T1D, and average HbA1c as covariates, a single SNP (rs1337791) was significantly associated with DN (OR = 0.74; CI: 0.57–0.97; P = 0.028; Table 2), however this was not maintained following correction for multiple testing. Haplotype analysis did not improve association over single marker analysis. Subgroup analyses on the basis of ESRD status showed no stronger association with ESRD compared to those calculated for DN. Follow-up evaluation of the top 4 significant associations in this study were not supported within the independent US GoKinD dataset (Table 3).

**Table 3:** Association analysis from the independent US Genetics of Kidneys in Diabetes study for the 4 most significant SNPs identified from this study.

Gene	SNP	Affymetrix 5.0 proxy SNP	r^2^	Alleles	P-Value	Odds Ratio (C.I.)
*LRP6*	rs2075241	rs16907810	0.95	[C/T]	0.96	0.99 (0.83–1.18)
*LRP5*	rs3736228			[T/C]	0.53	1.06 (0.87–1.29)
*LRP5*	rs491347	rs576118	1	[G/A]	0.61	1.04 (0.88–1.22)
*LRP6*	rs13377971	rs11054710	1	[A/G]	0.32	0.89 (0.71–1.12)

These data were extracted from publicly available data on dbGAP (http://www.ncbi.nlm.nih.gov/gap, dataset phs000018.v2) and are based on a stratified analysis of 935 cases and 944 controls [12]. The genotyping was performed on the Affymetrix 5.0 SNP array. Although only one of the most significant SNPs identified from this study was genotyped directly on this platform, surrogate markers in high LD based on 1000 Genomes pilot data (http://www.1000genomes.org/), were used as proxies for the remaining 3 SNPs.

Having no prior hypotheses about interactions between SNPs within the genes analysed in this study, we assumed a more stringent level of significance (P<0.01) in assessing interactions. No evidence supporting interaction was observed (Table 4 and Table S1).

**Table 4:** Assessment of gene-gene pair-wise interactions.

SNP	rs13377971	rs2075241	rs3736228
**rs13377971**			
**rs2075241**	0.026		
**rs3736228**	0.790	0.459	
**rs491347**	0.064	0.633	0.205

P values for gene-gene interactions were obtained between the four most significant SNPs using likelihood ratio χ^2^ tests in the logistic regression. None attained significance at the P<0.01 level.

## Discussion

Expansion of the mesangium is a well recognised histological feature of DN that occurs early in renal dysfunction. Lin and colleagues [2] showed that reduced expression of the endogenous inhibitor, DKK-1, which binds Wnt co-receptors LRP5/6, decreased diabetes-induced glomerular injury preventing hyperglycaemia-induced mesangial cell dysfunction. This homeostatic interaction between β-catenin and DKK-1 in mesangial cell function may result in variable β-catenin activity under different physiological and pathological conditions in renal tissue. In addition, they reported modulation of DKK-1, TGF-β1, and fibronectin expression through RNA interference suggesting a novel potential therapeutic target for DN [2].

Our study focused on the canonical Wnt pathway although other Wnt pathways exist, including Wnt/Ca^2+^ pathway and the planar cell polarity pathway which could also conceivably contribute to the pathogenesis of DN. The genes assessed in this study were chosen on the basis of gene expression profiles derived from human kidney biopsy samples of DN (unpublished data). Although the four genes investigated did not demonstrate significant association following correction for multiple testing, independent replication or provide evidence of significant interaction, additional untested variants within the canonical and non-canonical Wnt pathways remain untested. To our knowledge, this is the first association study in DN to assess common variation within key genes regulating the Wnt pathway. Interestingly, novel splice variants were proposed to modulate Wnt pathway gene expression through mRNA stability and enhanced translational efficiency [13]. Since our study focused only on common variants, untyped, highly penetrant rare variants within these genes could also be associated with DN. Based on sample sizes used here and considering Bonferroni correction for 31 comparisons, our study provides 90% power to identify an allele with an odds ratio of 1.69, 1.50, 1.44 and 1.42 whose frequency in controls is 10%, 20%, 30% and 40% respectively. However, this study has insufficient power to detect effect sizes of smaller magnitude with odds ratios of 1.2/1.3 which are more often seen in common complex diseases (Table 5). Other factors such as copy number variation or epigenetic mechanisms (e.g. DNA methylation, microRNAs) may alter gene function affecting these pathways, contributing to disease risk.

**Table 5:** Study power to detect various odds ratios for selected minor allele frequencies.

		Minor Allele Frequency (MAF)			
		0.10	0.20	0.30	0.40
**Odds ratio**	1.2	29%	48%	59%	64%
	1.3	55%	80%	88%	92%
	1.4	78%	95%	98%	99%
	1.5	92%	99%	100%	100%

Power calculations are based on 650 cases and 700 controls with odds ratio ranging from 1.2–1.5 for SNPs with a MAF between 0.10 and 0.40 with no adjustment for multiple testing. Corresponding figures after adjustment are given in the Discussion.

In conclusion, we found no strong association between common variants in the *CTNNB1, AXIN2, LRP5*, and *LRP6* genes and DN. Further work to investigate other members of the Wnt/β-catenin or non-canonical pathways may identify potential risk factors for genetic susceptibility to DN.

## Supporting Information

Table S1 **Assessment of gene-gene pair-wise interactions.** P values for gene-gene interactions were calculated from likelihood ratio χ^2^ tests in the logistic regression with adjustment for centre, gender, duration of type 1 diabetes and HbA1c. Three attained significance at the P<0.01 level and are underlined. Were the comparisons to have been independent then four would have been expected to attained significance at the P<0.01 level by chance purely as a consequence of multiple testing.
(DOC)
