# Supplementary material for: Association Analysis of Canonical Wnt Signalling Genes in Diabetic Nephropathy
Source: PLoS One. 2011 Aug 18;6(8):e23904. doi: 10.1371/journal.pone.0023904 (PMC3158097; doi:10.1371/journal.pone.0023904)
Supplement: Table S1 — Assessment of gene-gene pair-wise interactions. P values for gene-gene interactions were calculated from likelihood ratio χ2 tests in the logistic regression with adjustment for centre, gender, duration of type 1 diabetes and HbA1c. Three attained significance at the P<0.01 level and are underlined. Were the comparisons to have been independent then four would have been expected to attained significance at the P<0.01 level by chance purely as a consequence of multiple testing. (DOC) [file pone.0023904.s001.doc]

| **Gene** | **SNP** | rs10466849 | rs11228202 | rs11564465 | rs11823032 | rs11868547 | rs12452196 | rs13377971 | rs2075241 | rs2240308 | rs2242340 | rs2300230 | rs2302685 | rs2417085 | rs312014 | rs312016 | rs3736228 | rs3741792 | rs3781600 | rs3923086 | rs4074947 | rs4128941 | rs4541111 | rs4791171 | rs491347 | rs4930573 | rs587397 | rs7224837 | rs7305037 | rs740026 | rs74744 |
| --- | --- | --- | --- | --- | --- | --- | --- | --- | --- | --- | --- | --- | --- | --- | --- | --- | --- | --- | --- | --- | --- | --- | --- | --- | --- | --- | --- | --- | --- | --- | --- |
| *LRP6* | rs10466849 |  |  |  |  |  |  |  |  |  |  |  |  |  |  |  |  |  |  |  |  |  |  |  |  |  |  |  |  |  |  |
| *LRP5* | rs11228202 | 0.23 |  |  |  |  |  |  |  |  |  |  |  |  |  |  |  |  |  |  |  |  |  |  |  |  |  |  |  |  |  |
| *CTNNB1* | rs11564465 | 0.76 | 0.25 |  |  |  |  |  |  |  |  |  |  |  |  |  |  |  |  |  |  |  |  |  |  |  |  |  |  |  |  |
| *LRP5* | rs11823032 | 0.96 | 0.59 | 0.74 |  |  |  |  |  |  |  |  |  |  |  |  |  |  |  |  |  |  |  |  |  |  |  |  |  |  |  |
| *AXIN2* | rs11868547 | 0.86 | 0.96 | 0.80 | 0.49 |  |  |  |  |  |  |  |  |  |  |  |  |  |  |  |  |  |  |  |  |  |  |  |  |  |  |
| *AXIN2* | rs12452196 | 0.69 | 0.81 | 0.49 | 0.19 | 0.55 |  |  |  |  |  |  |  |  |  |  |  |  |  |  |  |  |  |  |  |  |  |  |  |  |  |
| *LRP6* | rs13377971 | 0.18 | 0.58 | 0.76 | 0.97 | 0.82 | 0.76 |  |  |  |  |  |  |  |  |  |  |  |  |  |  |  |  |  |  |  |  |  |  |  |  |
| *LRP6* | rs2075241 | 0.003 | 0.07 | 0.23 | 0.23 | 0.27 | 0.54 | 0.03 |  |  |  |  |  |  |  |  |  |  |  |  |  |  |  |  |  |  |  |  |  |  |  |
| *AXIN2* | rs2240308 | 0.54 | 0.61 | 0.51 | 0.61 | 0.50 | 0.83 | 0.64 | 0.92 |  |  |  |  |  |  |  |  |  |  |  |  |  |  |  |  |  |  |  |  |  |  |
| *LRP5* | rs2242340 | 0.88 | 0.06 | 0.65 | 0.86 | 0.29 | 0.47 | 0.66 | 0.73 | 0.07 |  |  |  |  |  |  |  |  |  |  |  |  |  |  |  |  |  |  |  |  |  |
| *LRP6* | rs2300230 | 0.60 | 0.08 | 0.83 | 0.11 | 0.48 | 0.28 | 0.18 | 0.10 | 0.16 | 0.11 |  |  |  |  |  |  |  |  |  |  |  |  |  |  |  |  |  |  |  |  |
| *LRP6* | rs2302685 | 0.34 | 0.57 | 0.70 | 0.03 | 0.31 | 0.94 | 0.69 | 0.07 | 0.92 | 0.08 | 0.29 |  |  |  |  |  |  |  |  |  |  |  |  |  |  |  |  |  |  |  |
| *LRP6* | rs2417085 | 0.03 | 0.48 | 0.42 | 0.17 | 0.13 | 0.91 | 0.67 | 0.53 | 0.88 | 0.56 | 0.28 | 0.27 |  |  |  |  |  |  |  |  |  |  |  |  |  |  |  |  |  |  |
| *LRP5* | rs312014 | 0.75 | 0.74 | 0.28 | 0.69 | 0.06 | 0.89 | 0.73 | 0.93 | 0.82 | 0.32 | 0.75 | 0.75 | 0.54 |  |  |  |  |  |  |  |  |  |  |  |  |  |  |  |  |  |
| *LRP5* | rs312016 | 0.37 | 0.47 | 0.20 | 0.22 | 0.11 | 0.73 | 0.68 | 0.28 | 0.61 | 0.32 | 0.87 | 0.70 | 0.30 | 0.05 |  |  |  |  |  |  |  |  |  |  |  |  |  |  |  |  |
| *LRP5* | rs3736228 | 0.55 | 0.32 | 0.88 | 0.47 | 0.76 | 0.78 | 0.79 | 0.46 | 0.92 | 0.58 | 0.30 | 0.95 | 0.73 | 0.06 | 0.06 |  |  |  |  |  |  |  |  |  |  |  |  |  |  |  |
| *LRP6* | rs3741792 | 0.37 | 0.07 | 0.82 | 0.08 | 0.51 | 0.45 | 0.11 | 0.05 | 0.20 | 0.08 | 0.90 | 0.32 | 0.42 | 0.72 | 0.67 | 0.34 |  |  |  |  |  |  |  |  |  |  |  |  |  |  |
| *LRP5* | rs3781600 | 0.32 | 0.50 | 0.63 | 0.23 | 0.82 | 0.64 | 0.86 | 0.14 | 0.99 | 0.16 | 0.29 | 0.54 | 0.42 | 0.80 | 0.65 | 0.76 | 0.25 |  |  |  |  |  |  |  |  |  |  |  |  |  |
| *AXIN2* | rs3923086 | 0.49 | 0.37 | 0.17 | 0.66 | 0.52 | 0.61 | 0.72 | 0.40 | 0.68 | 0.19 | 0.31 | 0.23 | 0.07 | 0.32 | 0.47 | 0.17 | 0.30 | 0.23 |  |  |  |  |  |  |  |  |  |  |  |  |
| *AXIN2* | rs4074947 | 0.97 | 0.86 | 0.63 | 0.69 | 0.38 | 0.66 | 0.50 | 0.93 | 0.42 | 0.76 | 0.76 | 0.52 | 0.14 | 0.92 | 0.51 | 0.86 | 0.78 | 0.73 | 0.26 |  |  |  |  |  |  |  |  |  |  |  |
| *AXIN2* | rs4128941 | 0.16 | 0.03 | 0.26 | 0.45 | 0.59 | 0.52 | 0.08 | 0.40 | 0.41 | 0.52 | 0.54 | 0.68 | 0.69 | 0.69 | 0.52 | 0.82 | 0.37 | 0.05 | 0.88 | 0.49 |  |  |  |  |  |  |  |  |  |  |
| *AXIN2* | rs4541111 | 0.84 | 0.47 | 0.73 | 0.72 | 0.20 | 0.15 | 0.70 | 0.37 | 0.37 | 0.34 | 0.61 | 0.28 | 0.12 | 0.16 | 0.36 | 0.91 | 0.65 | 0.29 | 0.99 | 0.86 | 0.98 |  |  |  |  |  |  |  |  |  |
| *AXIN2* | rs4791171 | 0.86 | 0.32 | 0.80 | 0.28 | 0.06 | 0.64 | 0.34 | 0.32 | 0.57 | 0.38 | 0.54 | 0.25 | 0.006 | 0.08 | 0.08 | 0.94 | 0.48 | 0.38 | 0.41 | 0.86 | 0.52 | 0.12 |  |  |  |  |  |  |  |  |
| *LRP5* | rs491347 | 0.08 | 0.04 | 0.82 | 0.98 | 0.62 | 0.83 | 0.07 | 0.63 | 0.21 | 0.15 | 0.11 | 0.97 | 0.30 | 0.05 | 0.03 | 0.21 | 0.07 | 0.06 | 0.61 | 0.85 | 0.96 | 0.84 | 0.96 |  |  |  |  |  |  |  |
| *LRP5* | rs4930573 | 0.58 | 0.61 | 0.74 | 0.35 | 0.41 | 0.44 | 0.08 | 0.40 | 0.39 | 0.89 | 0.32 | 0.48 | 0.35 | 0.72 | 0.84 | 0.05 | 0.21 | 0.98 | 0.83 | 0.29 | 0.82 | 0.71 | 0.56 | 0.80 |  |  |  |  |  |  |
| *LRP5* | rs587397 | 0.04 | 0.18 | 0.91 | 0.28 | 0.80 | 0.82 | 0.009 | 0.37 | 0.15 | 0.11 | 0.41 | 0.76 | 0.29 | 0.51 | 0.69 | 0.03 | 0.21 | 0.21 | 0.50 | 0.62 | 0.38 | 0.72 | 0.99 | 0.39 | 0.35 |  |  |  |  |  |
| *AXIN2* | rs7224837 | 0.31 | 0.21 | 0.55 | 0.70 | 0.10 | 0.53 | 0.14 | 0.70 | 0.91 | 0.76 | 0.81 | 0.25 | 0.34 | 0.02 | 0.01 | 0.50 | 0.88 | 0.24 | 0.72 | 0.09 | 0.94 | 0.29 | 0.40 | 0.86 | 0.40 | 0.94 |  |  |  |  |
| *LRP6* | rs7305037 | 0.44 | 0.66 | 0.99 | 0.15 | 0.13 | 0.47 | 0.93 | 0.73 | 0.63 | 0.47 | 0.09 | 0.99 | 0.96 | 0.45 | 0.15 | 0.47 | 0.30 | 0.48 | 0.03 | 0.52 | 0.88 | 0.14 | 0.07 | 0.14 | 0.56 | 0.18 | 0.25 |  |  |  |
| *AXIN2* | rs740026 | 0.63 | 0.74 | 0.10 | 0.83 | 0.31 | 0.85 | 0.21 | 0.54 | 0.09 | 0.10 | 0.03 | 0.97 | 0.11 | 0.87 | 0.55 | 0.99 | 0.06 | 0.70 | 0.29 | 0.08 | 0.35 | 0.15 | 0.20 | 0.62 | 0.26 | 0.30 | 0.94 | 0.73 |  |  |
| *LRP5* | rs74744 | 0.94 | 0.52 | 0.68 | 0.43 | 0.58 | 0.36 | 0.96 | 0.22 | 0.99 | 0.32 | 0.22 | 0.05 | 0.41 | 0.16 | 0.06 | 0.79 | 0.21 | 0.97 | 0.50 | 0.86 | 0.25 | 0.35 | 0.10 | 0.70 | 0.47 | 0.88 | 0.37 | 0.40 | 0.42 |  |
| *AXIN2* | rs757558 | 0.16 | 0.64 | 0.05 | 0.35 | 0.29 | 0.36 | 0.69 | 0.97 | 0.41 | 0.60 | 0.14 | 0.21 | 0.70 | 0.71 | 0.97 | 0.67 | 0.21 | 0.93 | 0.33 | 0.71 | 0.76 | 0.25 | 0.78 | 0.63 | 0.13 | 0.75 | 0.47 | 0.15 | 0.45 | 0.80 |

**Supplementary table 1.**  Assessment of gene-gene pair-wise interactions. P values for gene-gene interactions were calculated from likelihood ratio χ^2^ tests in the logistic regression with adjustment for centre, gender, duration of type 1 diabetes and HbA1c. Three attained significance at the P<0.01 level and are underlined. Were the comparisons to have been independent then four would have been expected to attained significance at the P<0.01 level by chance purely as a consequence of multiple testing.
